# Developing Ecological Thresholds for Nitrogen and Phosphorus in the Haihe River Basin in China

**DOI:** 10.3390/ijerph192416951

**Published:** 2022-12-16

**Authors:** Fan Wu, Yuan Fang, Mingfeng Feng, Zhicheng Xie, Lin Zhu, Jianfeng Feng

**Affiliations:** 1 Jinan Environmental Research Academy, Jinan 250000, China; 2 Key Laboratory of Pollution Process and Environmental Criteria of Ministry of Education and Tianjin Key Laboratory of Environmental Remediation and Pollution Control, College of Environmental Science and Engineering, Nankai University, Tianjin 300071, China; 3 Tianjin Huanke Environment Consulting Co., Ltd., Tianjin 300450, China; 4 Tianjin Academy of Ecological and Environmental Science, Tianjin 300191, China

**Keywords:** total nitrogen, total phosphorus, thresholds, frequency distribution, TITAN

## Abstract

Many aquatic ecosystems are eutrophicated due to human inputs of nitrogen and phosphorus. Therefore, it is now considered important to establish nutrient criteria to prevent eutrophication. In this study, the water quality and biological data for 26 stations in the lower reaches of the Haihe River Basin were collected. The total nitrogen (TN) and total phosphorus (TP) ecological thresholds were derived from the threshold indicator taxa analysis (TITAN) and frequency distribution approach. The results showed that the TN threshold was 1.8 mg/L based on the TITAN and the TP threshold was 0.039 mg/L based on the frequency distribution approach. The TITAN also identified 29 indicator species of TN and 28 indicator species of TP. Based on these criteria, we found 73% sites with TN concentrations higher than the 1.8 mg/L. As for TP, 58% sites exceeded 0.039 mg/L. This study showed that most of the downstream areas of the Haihe River are subject to relatively serious disturbances. This finding could provide implications for identifying the water quality traits of and generating protection strategies for the Haihe River in Tianjin.

## 1. Introduction

The input of nutrients to aquatic ecosystems has been highly increased due to human activities, leading to severer eutrophication, which has become a non-ignorable problem facing aquatic ecosystems in China [1,2,3].

According to Groffman et al. [4], when the physical and chemical characteristics of an ecosystem or the structure and function of a biological community suddenly change dramatically due to a small change in one or several environmental factors in the ecosystem, the point corresponding to this one or several environmental factors is the ecological threshold. This means that there are thresholds or breakpoints between different states of the ecosystem. When the external interference is too strong, beyond the scope of the ecosystem, the community structure of the ecosystem will also change dramatically, which may lead to the destruction or even loss of the original functions of the ecosystem.

River ecosystems will degenerate when the water quality fails to reach the assigned ecological criteria. The nutrient criteria are needed to help regulators making efforts to protect aquatic ecosystems from eutrophication. Furthermore, the nutrient criteria are used to set specific concentration goals [5,6,7], which will help researchers to propose clear management measures.

Phytoplankton are the largest source of primary production, whose community composition can directly and quickly reveal the dynamic changes in water environments in aquatic ecosystems [4]. Phytoplankton communities, which play a crucial part in trophic transfer through food chain [8], can be used to develop nutrient criteria for river ecosystems because nitrogen and phosphorus are the main materials required in the process of phytoplankton growth and development [9]. The TN and TP have a significant influence on the total biomass and structure of phytoplankton communities [10,11,12,13] and play a vital role in assessing the eutrophication degree of water bodies [14,15].

In this study, phytoplankton, as response taxa to nutrient variation, were selected to develop the thresholds for TN and TP in the downstream areas of the Haihe River Basin, where the ecological environment has seriously deteriorated due to eutrophication. We collected data on the planktonic algae and water quality in October 2017 at 26 locations in Tianjin. The main purpose of this study is to reveal the water quality status in the surveyed area and establish thresholds for TN and TP based on a TITAN to provide guidelines for the management of the Haihe River Basin.

## 2. Materials and Methods

### 2.1. Study Sites and Field Sampling

**Figure 1:**
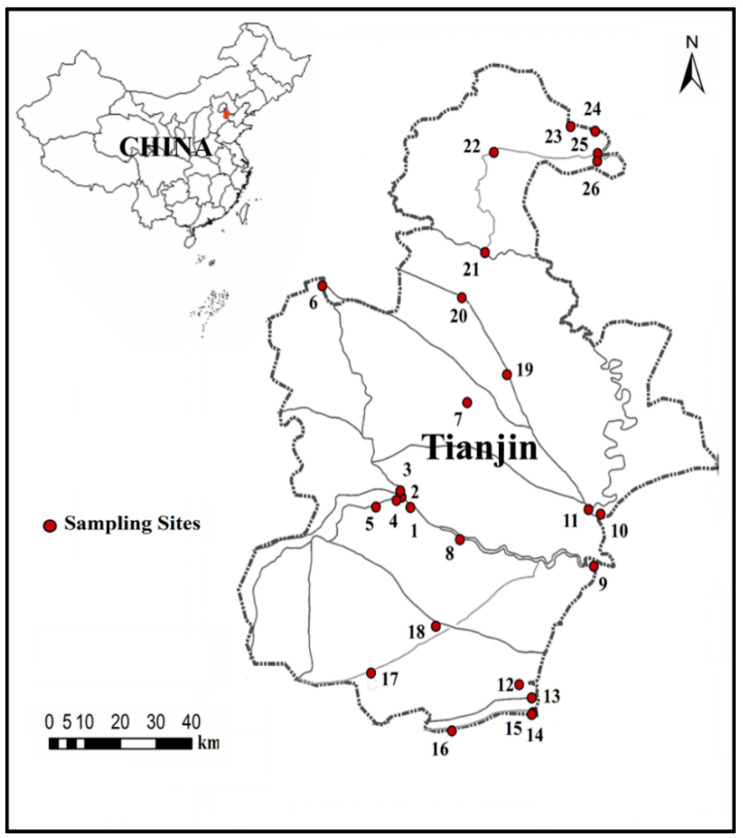
Locations of sampling sites in Tianjin City in China.

The Haihe River Basin has a watershed area of 318,000 square kilometers. As the largest water system in North China, it plays an important role in contributing to the economic and social development of North China. The lower reaches of this area are located at the junction of the North China Plain and the Bohai Sea. There are many developed cities and strong human activities in this area [16]. Over the past few decades, these human activities have greatly affected the levels of nutrients in the basin, resulting in substantial increases in nitrogen and phosphorus in the Haihe River Basin and reductions in aquatic biodiversity. During October 2017, we collected samples from 26 sites distributed throughout Tianjin City for phytoplankton, zooplankton, and water quality measurements (Figure 1). The samples were collected using the methods described in Water Quality Guidance on Sampling Techniques for Rivers [17].

### 2.2. Water Quality Analysis

The total nitrogen (TN), total phosphorus (TP), ammonia nitrogen (NH_4_^+^), nitrate nitrogen (NO_3_^−^), nitrite nitrogen (NO_2_^−^), phosphate (PO_4_^3−^), and permanganate index (COD_Mn_) levels were analyzed according to the method given in the State Environmental Protection Administration guidance [18] (see Table S1 from Supplementary Materials).

### 2.3. Biodiversity Index Analysis

The identification of phytoplankton and zooplankton taxa were conducted according to the methods used by Hu and Wei (2006) [19] and Zhou and Chen (2010) [20], respectively, and both groups were classed into species. We calculated the average density of each species at each site. The Shannon–Wiener diversity index(H), Pielou evenness index(J), and Margalef richness index(D) were calculated according to Rosenzweig (1976) [21]. The three biodiversity indexes were calculated as follows:

$$\mathrm{Shannon}\text{-}\mathrm{Wiener}\;\mathrm{index}\;=-\sum_{i=1}^{s}p_{i}\log p_{i}$$

$$\mathrm{Margalef}\;\mathrm{richness}\;\mathrm{index}=\frac{S-1}{\log_{2}N}$$

$$\mathrm{Pielou}\;\mathrm{evenness}\;\mathrm{index}=\frac{H}{\log_{2}S}$$

where *p* is the proportion (n/N) of individuals of one particular species (n) divided by the total number of individuals found (N), S is the number of species, and H is Shannon‘s diversity index.

### 2.4. Data Analysis

In this study, the canonical correspondence analysis (CCA) was used to summarize a data set and to evaluate the relationships between the phytoplankton’s community assemblage and their environmental variables while considering that the length of the first gradient in the detrended correspondence analysis (DCA) was greater than 4. The multicollinearity diagnosis can be performed using the variance inflation factor (VIF). Collinear variables with VIF > 10 were left out the CCA analysis. The VIFs were calculated in R using the vif.cca() function in the vegan library [22] The VIFs of all water quality variables are shown in Tables S2 from Supplementary Materials. Pearson’s correlation analysis was used to explore the relationship between phytoplankton diversity indexes and environmental variables. All analyses were performed in R.3.5.2(Kurt Hornik and the R Core Team, 2022) in the vegan library.

It has been shown that threshold indicator taxa analysis (TITAN) is an effective approach for identifying indicator species and establishing ecological thresholds [23,24]. This approach combines an indicator species analysis [25] and change point analysis (nCPA) [26] to determine indicator values for each candidate change point along the stressor gradient and then uses bootstrapping to identify reliable indicator taxa. Before conducting the TITAN analysis, log10 (x + 1) transformations were performed on species abundances and the species with <3 occurrences were excluded to reduce the impact on indicator value calculations brought on by rare species with low abundance rates in the present study. In the nCPA, the data for the sampling sites were split into two groups along a nutrient gradient to produce optimal change points, indicating the greatest differences in the mean and variance within the groups [27]. We used the purity and reliability as diagnostic indexes to identify indicator species. In this study, we set only the reliability (≥0.95) and purity (≥0.95) of the taxa as the minimum requirements. More information about the TITAN is shown by Baker and King (2010) [28].

## 3. Results

### 3.1. Relationship between Water Quality Indicators and Phytoplankton Taxa

**Figure 2:**
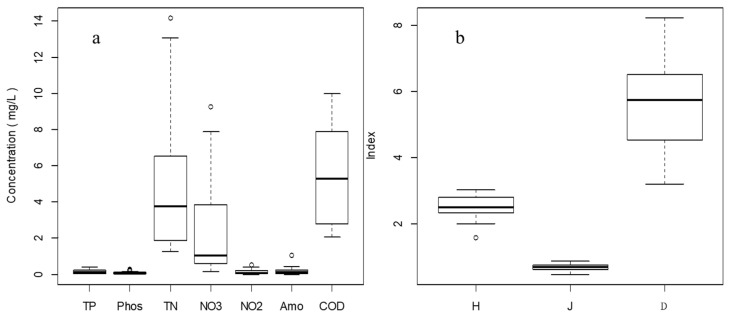
The water chemistry (**a**) and phytoplankton community structure parameters (**b**) for the Haihe River in Tianjin, China. H, J, and D represent the Shannon–Wiener index, Pielou evenness index, and Margalef richness index values, respectively, for the Haihe River in Tianjin, China.

In the downstream areas of the Haihe River Basin, the sampling sites revealed relatively high concentrations of nutrients. The TN concentrations ranged from 1.26 to 14.17 mg/L and the range for the TP values was from 0.013 to 0.379 mg/L. The NH_4_^+^ concentrations ranged from 0.002 to 1.03 mg/L and the concentrations ranged from 0.01 to 0.28 mg/L for PO_4_^3−^. The concentration ranges for NO_2_^−^, NO_3_^−^, and COD_Mn_ were from 0.002 to 0.53, from 0.13 to 9.27, and from 2.06 to 9.98 mg/L, respectively. The density levels of phytoplankton assemblages varied from 1.28 × 10^6^ to 6.37 × 10^7^ cell per liter. The Shannon–Wiener diversity index, Pielou evenness index, and Margalef richness index values ranged from 1.58 to 3.04, from 0.49 to 0.87, and from 3.22 to 8.23, respectively (Figure 2).

**Figure 3:**
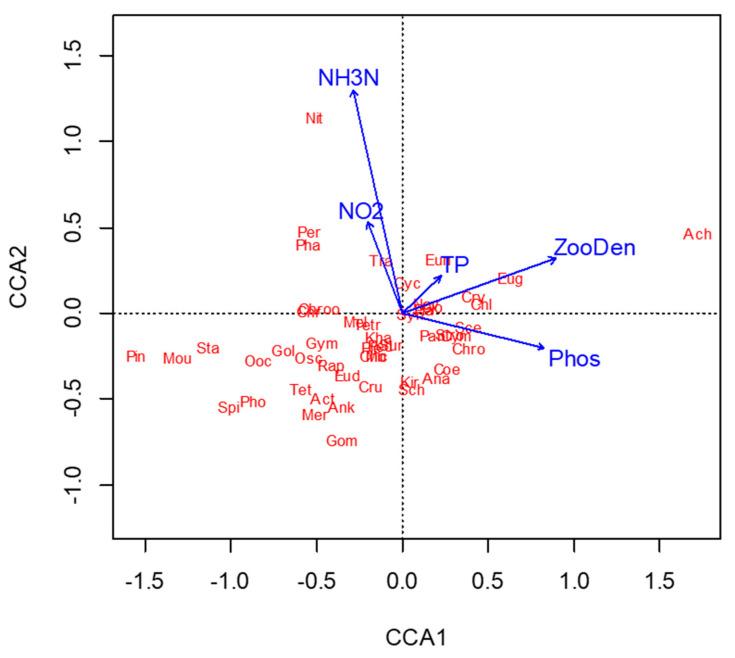
CCA ordination diagram of the environmental variables (TN, TP, NH_4_^+^, NO_3_^−^, NO_2_^−^, PO_4_^3−^, COD_Mn_), zooplankton density (ZooDen), and phytoplankton community (see Table S3 for details of the phytoplankton taxa).

The CCA analysis showed there were 5 water chemistry parameters explaining 43.87% of the total variation in the phytoplankton community (Figure 3), along with the Monte Carlo permutation test result (F = 1.55, *p* < 0.05). The NH_4_^+^, PO_4_^3−^, and zooplankton density levels are the important factors affecting the distribution of phytoplankton (Figure 3). A total of 50 phytoplankton genera were found in 26 sampling sites in the downstream of the Haihe River Basin and are listed in Table S3 from Supplementary Materials. Pearson’s correlation analysis showed that there were significant negative correlations between the TP and the three indexes (i.e., Shannon–Wiener diversity index, Pielou evenness index, and Margalef richness index), indicating that the TP might have an important impact on the diversity of the phytoplankton (*p* < 0.01). In contrast, the TN and NO_3_^−^ showed weak correlations with three indexes (see Table S4 from Supplementary Materials). Additionally, the Shannon–Wiener diversity index, Pielou evenness index, and Margalef richness index correlated perfectly with one another, while the interrelationships of the TP, PO_4_^3−^, and COD_Mn_ and the NO_2_^−^ and NH_4_^+^ were also significant (*p* < 0.01) (see Tables S4 from Supplementary Materials).

### 3.2. Threshold Responses to Nutrients and Indicator Taxa

**Figure 4:**
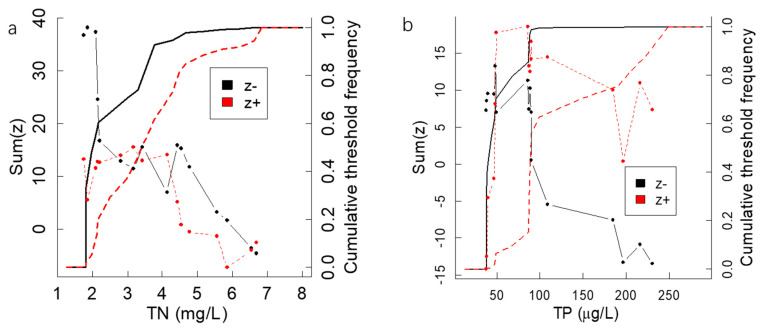

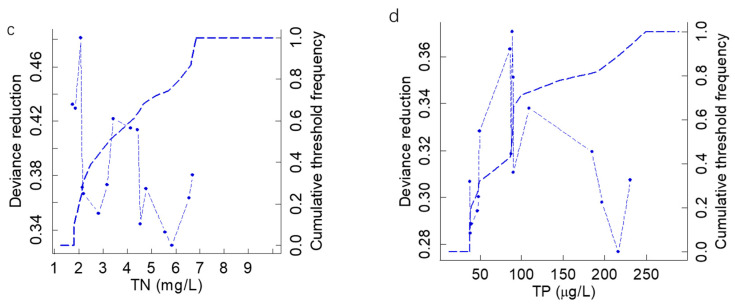
The TITAN sums of negative (z−) and positive (z+) responding species to all candidate change points along the TN and TP gradients (**a**,**b**). The deviance reduction in the Bray–Curtis distance values for the candidate change points of the phytoplankton community along TN and TP gradients and the cumulative frequency distribution of the change points among the bootstrap replicates (**c**,**d**). The dotted lines respectively represent the responses of negative responding species (z− ) and positive responding species (z+) along the nutrient gradients, while the broken lines represent the cumulative frequency distribution of the change points among the bootstrap replicates.

The results of the TITAN showed that a sum (z+) change point was found at 3.30 mg/L along the TN concentration gradients, whereas a sum (z−) change point was found at 1.80 mg/L. The sum (z+) reached a peak at 87 µg/L along the TP concentration gradient, whereas the sum (z−) peaked at 49 µg/L (Figure 4). Figure 4 shows that the phytoplankton community reaches the negative response threshold when the TN and TP are at lower concentrations, while the sum (z+) peaks are not very distinct along the TN and TP concentration gradients.

**Figure 5:**
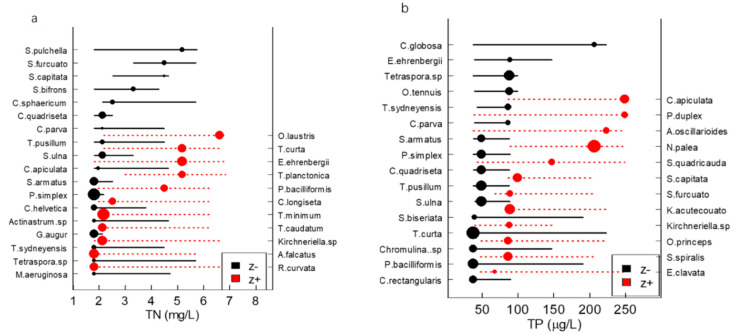
The TITAN results showing significant indicator taxa of TN and TP (**a**,**b**). The circles show taxa change points, which were sized according to the magnitudes of response.

There were 29 indicator species obtained using the TITAN for the TN (18 of which were negative response species, while 11 were positive response species) and 28 indicator species for the TP (16 of which were negative response species and 12 were positive response species). Among the TN indicator species, the Chlorophyta accounted for the largest proportion (50% of the negative indicator species and 54.54% of the positive ones), followed by Bacillariophyta and Euglenophyta, accounting for 38.89% and 36.36% of the negative indicator species and positive indicator species, respectively, while also representing the largest proportions of Chlorophyta among the TP indicator species, at 43.75% and 50% for negative and positive indicator species, respectively. *Tetraedron pusillum*, *Chodatella quadriseta*, *Tetraspora* sp., *Pediastrum simplex*, *Scenedesmus armatus*, *Trachelomonas sydneyensis*, *Synedra ulna,* and *Cymbella parva* appeared in the TN and TP negative indicator species, whereas only *Kirchneriella* sp. Appeared in the TN and TP positive indicator species (Figure 5).

## 4. Discussion

Nutrient criteria, as important references for water pollution control, are useful for assisting regulators to control the eutrophication of water bodies, assess the impacts of human activities on river ecosystems, and protect the biodiversity of aquatic ecosystems [29]. They are used as crucial tools for protecting and restoring water bodies from nitrogen and phosphorus pollution. The nutrient criteria can be used to establish a threshold for a pollutant or condition, indicating that the water body may be threatened when the water quality is above or below the designated threshold [30]. They are the critical basis for developing ecological-threshold-related policies in aquatic ecosystems [31].

### 4.1. Water Quality Indicators and Phytoplankton Taxa

As the results showed above, the TP, PO_4_^3−^, and NO_2_^−^ have greater effects on the distribution of phytoplankton communities, and TP and PO_4_^3−^ have especially significantly impacts on phytoplankton density (*p* < 0.01). Although the CCA shows the relationship between the species structure and the environmental parameters, it cannot effectively solve the problems of collinearity among environmental factors [32]. In the future, more environmental information should be considered in research to provide a deeper and more comprehensive environmental explanation.

### 4.2. Nutrient Thresholds

There are many methods that can be used to evaluate nutrient criteria based on the data quality and study area (see Table S5 from Supplementary Materials). Charles et al. (2019) [33] identified potential nutrient criteria using a new approach, the biological condition gradient (BCG). The BCG levels reveal the gradient of ecological conditions from natural to highly impaired, and a large gradient of nutrient concentrations was required for the study area. Chen et al. (2018) [34] evaluated the nutrient criteria of streams and rivers in the Qing River system by applying the reference stream distribution approach, all-streams distribution approach, and Y-intercept approach, the values of which were 0.724–1.288 mg/L for TN and 0.024–0.046 mg/L for TP. The reference stream distribution approach may not be widely applicable, as there are usually few reference conditions in the developed region [35]. Nutrient thresholds based on the all-streams frequency distribution approach usually lead to over- or underprotection [36]. The Y-intercept approach primarily focuses on several factors affecting water quality. However, water quality depends on various factors [37]. In contrast, the stress–response approach allows the identification of biological responses, such as the CART, nCPA, BHM, and TITAN values. Liu et al. (2018) [38] determined the nutrient criteria of lakes and reservoirs in Heilongjiang Province, China, using the CART, nCPA, and BHM models, finding no significant differences among the numerical nutrient criteria. Those approaches based on the stress–response relationship do not require the collection of a large amount of data from reference or minimally impacted conditions, and can reflect the actual situation of the water body and be used to comprehensively consider the ecological characteristics of the study area to provide more scientific and reasonable criteria [39]. In this study, the TN and TP thresholds evaluated according to the TITAN approach were 1.800 mg/L and 0.049 mg/L, respectively.

In addition, the TN threshold was higher than those in other studies (0.382–1.288 mg/L), This result may have been because the load of nitrogen in the Tianjin section of the Haihe River is high, while the degree of eutrophication is relatively serious, with inferior V [40] water function zones accounting for more than 25% of the area according to the Tianjin Ecology and Environment Statement in 2018.

The TITAN identified ecological community thresholds from multiple change points. The TN peak occurred at the lower concentrations, which indicated that most of the negative response taxa occur abruptly and with synchronous changes. The responses of the negative indicators to TN were much more abrupt and synchronous, ranging from about 1.0 to 2.5mg/LTN. The strongest threshold indicators responded quite synchronously, with the values ranging between 1.5 and 2.0 mg/L of TN, as evidenced by the clustering of larger filled symbols at low levels of TN (Figure 5a). In other words, many taxa are suitable in water bodies with low TN concentrations. When the TN increased, the density or abundance of the sensitive species declined significantly. Neither Figure 5a or Figure 5b show a clear crossover point, which might show that the pattern of many taxa increasing and decreasing in a relatively narrow range of the gradient is not evident. In other words, most phytoplankton taxa have strong tolerances within the measured TP and TN concentration ranges, which leads to no distinct z(+) peaks. This may be because the sampling points are mainly distributed in the lower reaches of the Haihe River Basin, meaning there is no obvious continuum of change in the middle of the gradient of TP and TP concentrations.

### 4.3. Indicator Species

On the one hand, the TITAN revealed the threshold range of positive and negative responses of the phytoplankton community, indicating that when the concentrations of TN and TP in the river are low, the sensitive species first respond to the nutrient change; with the increase in nutrient levels, the biomass and diversity of the phytoplankton community are affected successively, and when the nutrient level increases to a certain extent, the density or abundance of tolerant species in the community will change significantly.

Among the negative TN indicator species, the Chlorophyta and Bacillariophyta accounted for the largest proportions (accounting for 50% and 38.89%, respectively), whereas Chlorophyta and Euglenophyta accounted for the largest proportions among the positive TN indicator species, at 54.55% and 36.36%, respectively. In this study, a z(+) species increase was regarded as an undesirable trend, considering that Euglenophyta is the main group in mesotrophic water bodies, whereas Bacillariophyta, preferring to live in low-carbon water bodies, can be seen as an indicator group for oligotrophic water bodies [41]. The proportion of Cyanophyta (16.67%) was higher in the positive TP indicator species as compared to 6.25% proportion in the negative TP indicator species. Cyanobacteria have strong adaptability and can grow rapidly in suitable environments, such as in October with higher water temperatures, and can become a dominant population, which may lead to water bloom and cause deterioration of the water quality [42].

Although various organisms have certain ranges of adaptation, other conditions such as the geography, climate, river bottom, flow rate, and water depth are also important for the survival and distribution of living things. When using indicator organisms to monitor and evaluate water quality, one must pay attention to these factors. In recent decades, the problem of eutrophication has become increasingly serious with the increasing intensification of human activities [43]. There is an urgent need to develop related nutrient criteria in order to reasonably determine TN and TP control standards and provide significant information for managing river ecosystems.

## 5. Conclusions

The results showed that it is useful to develop nutrient criteria to effectively control excessive nutrient loading. The nutrient criteria for the TN and TP are suggested seriously to be no more than 3.30 mg/L and 0.087 mg/L, respectively. Furthermore, the nutrient concentrations should preferably be controlled below 1.80 mg/L TN and 0.039 mg/L of TP, respectively, to maintain the naturally aquatic integrity of ecosystems in the downstream areas of the Haihe River. In summary, this is an important area of future research to explore the potential influence of nutrient enrichment on phytoplankton communities, which will contribute to developing effective management regulations related to ecosystem protection and restoration.

## Data Availability

The data for this study are available from the corresponding author upon reasonable request.

## Supplementary Materials

The following supporting information can be downloaded at: https://www.mdpi.com/article/10.3390/ijerph192416951/s1. Table S1. Methods of water quality analysis. Table S2. Variance inflation factors (VIFs) of water quality variables. Table S3. Phytoplankton taxa recorded from 26 sampling sites. Table S4. The correlation between water quality parameters and phytoplankton indexes. Table S5. Nutrient criteria for TN and TP from the literature. References [18,22] are added in Supplementary file.
