# Supplementary material for: Developing Ecological Thresholds for Nitrogen and Phosphorus in the Haihe River Basin in China
Source: Int J Environ Res Public Health. 2022 Dec 16;19(24):16951. doi: 10.3390/ijerph192416951 (PMC9779712; doi:10.3390/ijerph192416951)
Supplement: Supplementary file 1 [file ijerph-19-16951-s001.zip › ijerph-2034215-supplementary (1)-done.pdf]

International Journal of
Environmental Research and Public Health

Table S1. Methods of water quality analysis.

Variables	Methods of Water Quality Analysis
TN	alkaline potassium persulfate digestion UV spectrophotometry
TP	Ammonium molybdate spectrophotometric method
NH3N	Spectrophotometry with salicylic acid
NO3	UV spectrophotometry
NO2	Diazamine coincidence spectrophotometry
Phos	molybdenum antimony anti-spectrophotometry;
COD_{Mn}	Potassium Permanganate titration

Details about the methods are described in State Environmental Protection Administration[1].

Table S2. Variance Inflation Factors (VIFs) of water quality variables.

Water Quality Variables	VIF
TP	8.3218
Phos	5.0012
TN	35.6425
NO3	38.5025
NO2	2.5957
NH_3N	7.8240
COD_{Mn}	12.5663
ZooDen	1.8180

Collinear variables with VIF>10 are left out the CCA analysis[2] (Karlsson, 2007).

Table S3. Phytoplankton taxa recorded from 26 sampling sites.

Code	Genus	Species
Ach	Achnanthes	*Achnanthes lanceolata*
Act	Actinastrum	*Actinastrum.sp*
Ana	Anabaena	*Anabaena circinalis*
		Anabaena oscillarioides
Ank	Ankistrodesmus	*Ankistrodesmus falcatus*
Chl	Chlamydomonas	*Chlamydomonas globosa*
Cho	Chodatella	*Chodatella longiseta*
		Chodatella quadriseta
Chr	Chromulina	*Chromulina .sp*
Chro	Chroococcus	*Chroococcus.sp*
Chroo	Chroomonas	*Chroomonas acuta*
Clo	Closterium	*Closterium acerosum*
		Closterium acutum
		Closterium gracile
Coe	Coelastrum	*Coelastrum sphaericum*
Cru	Crucigenia	*Crucigenia apiculata*
		Crucigenia rectangularis
		Crucigenia tetrapedia
Cry	Cryptomons	*Cryptomons ovata*
Cyc	Cyclotella	*Cyclotella comensis*
		Cyclotella meneghiniana
Cym	Cymbella	*Cymbella gracilis*
		Cymbella helvetica
		Cymbella parva
		Cymbella pusilla
Eud	Eudorina	*Eudorina elegans*
Eug	Euglena	*Euglena clavata*
		Euglena ehrenbergii
		Euglena virids
Eun	Eunotia	*Eunotia arcus*
Gol	Golenkinia	*Golenkinia radiata*
Gom	Gomphonema	*Gomphonema augur*
		Gomphonema parvulum
		Gomphonema simus
Gym	Gymnodinium	*Gymnodinium aerucyinosum*
Kha	Khawkinea	*Khawkinea acutecouato*
Kir	Kirchneriella	*Kirchneriella.sp*

Mel	Melosira	*Melosira granulata*
Mer	Merismopedia	*Merismopedia punctata*
Mic	Microcystis	*Microcystis aeruginosa*
		Microcystis marginata
		Microcystis robusta
Mou	Mougeotia	*Mougeotia scalaris*
Nav	Navicula	*Navicula avenacea*
		Navicula cincta
		Navicula cuspidata
		Navicula exigua
		Navicula viridula
Nit	Nitzschia	*Nitzschia palea*
		Nitzschia acicularis
		Nitzschia longissima
Ooc	Oocystis	*Oocystis laustris*
Osc	Oscillatoria	*Oscillatoria princeps*
		Oscillatoria tenuis
Pan	Pandorina	*Pandorina morum*
Ped	Pediastrum	*Pediastrum duplex*
		Pediastrum simplex
Per	Peridinium	*Peridinium umbonatum*
Pha	Phacus	*Phacus bacilliformis*
		Phacus circulatus
Pho	Phormidium	*Phormidium.sp*
Pin	Pinnularia	*Pinnularia acrosphaeria*
Pla	Platymonas	*Platymonas subcordiformis*
Ple	Pleodoria	*Pleodoria californica*
Rap	Raphidiopsis	*Raphidiopsis curvata*
Sce	Scenedesmus	*Scenedesmus armatus*
		Scenedesmus bicaudatus
		Scenedesmus bijuga
		Scenedesmus dimorphus
		Scenedesmus furcuato
		Scenedesmus quadricauda
Sch	Schroederia	*Schroederia spiralis*
Spi	Spirulina	*Spirulina platensis*
Sta	Staurastrum	*Staurastrum planctonicum*
Stro	Strombomonas	*Strombomonas fluviatilis*
Sur	Surirella	*Surirella bifrons*
		Surirella biseriata
		Surirella ovata
		Surirella tenera
Syn	Synedra	*Synedra capitata*
		Synedra pulchella
		Synedra ulna
Tet	Tetraedron	*Tetraedron caudatum*
		Tetraedron minimum
		Tetraedron pusillum
Tetr	Tetraspora	*Tetraspora.sp*
Tra	Trachelomonas	*Trachelomomas curta*
		Trachelomonas planctonica
		Trachelomonas sydneyensis

Table S4. The correlation between water quality parameter and phytoplankton indexes.

	**TP**	**Phos**	**TN**	**NO_3**	**NO_2**	**NH_3N**	**COD_{Mn}**	**H**	**J**	**D**	**ZooDen**
TP	1.00										
PO_4^{3-}	0.79**	1.00									
TN	0.15	-0.03	1.00								
NO_3^-	-0.16	-0.16	0.82**	1.00							
NO_2^-	0.36*	0.37*	0.40*	0.33	1.00						
NH_4^+	0.48*	0.06	0.49*	0.11	0.48**	1.00					
COD_{Mn}	0.63**	0.43**	0.17	-0.31	0.13	0.34*	1.00				
H	-0.36**	-0.29**	-0.09	-0.01	-0.32**	-0.09*	-0.30**	1.00			
J	-0.31**	-0.19*	0.06	0.08	-0.34**	-0.14*	-0.17*	0.89**	1.00		
D	-0.53**	-0.54**	-0.25	-0.03	-0.25*	-0.12	-0.60**	0.57**	0.25*	1.00	
ZooDen	0.07	0.06	0.06	0.09	0.31	0.14	0.16	-0.06	-0.18	-0.13	1.00

H: Shannon-Wiener index; J: Pielou evenness index; D: Margalef richness index; ZooDen: Zooplankton density; *p<0.05; **p<0.01.

Table S5. Nutrient criteria of TN and TP from some literatures.

Approach	TN (mg /L)	TP (mg/ L)	Study Area	Response Variable	Reference
BCG	1.000	0.050	streams in New Jersey，US	diatom	(Charles et al., 2019)
Y-intercept approach	0.749	0.035	Qing River，China		(Chen et al., 2018)
Frequency distribution[a]	1.288	0.046			
Frequency distribution[b]	0.724	0.024			
CART	0.725	0.058	lakes and reservoirs in Heilongjiang Province，China	Chl a	(Liu et al., 2018)
nCPA	0.865	0.058			
BHM	0.828	0.056			
TITAN	0.450	0.019	Lake Dianchi, China	periphytic diatom	(Cao et al., 2017)
TITAN	0.960	0.050	Yangtze connected lakes, China	diatom	(Zhang et al., 2016)
	0.520	0.020	Yangtze isolated lakes, China		
TITAN	0.382	0.016	Three Gorges Reservoir,China	epilithic diatom	(Tang et al., 2016)
TITAN	1.800	0.049	Haihe River in Tianjin, China	phytoplankton	This study

BCG: Biological Condition Gradient; CART: Classification and regression tree analysis. nCPA: Non-parametric change point analysis; BHM: Bayesian hierarchical model; [a]: Reference Stream Distribution Approach; [b]: All-Streams Distribution Approach.

References:

1. State Environmental Protection Administration1. Determination methods for examination of water and wastewater(fourth edition)[M].Beijing: Environmental Press of China,2002

2. Karlsson, A., Introduction to linear regression analysis. *Journal of the Royal Statistical Society Series a-Statistics in Society* **2007,** *170*, 856-857.
